# Clinical values of oblique lumbar interbody fusion on the treatment of single-level degenerative lumbar diseases

**DOI:** 10.3389/fsurg.2024.1424262

**Published:** 2024-09-05

**Authors:** Yu Yu

**Affiliations:** Department of Orthopedics, The Second People’s Hospital of Hefei, Hefei, China

**Keywords:** OLIF, Mis-TLIF, LBP, single-level degenerative lumbar disease, minimally invasive techniques

## Abstract

**Objectives:**

Minimally invasive transforaminal lumbar interbody fusion (Mis-TLIF) and oblique lumbar interbody fusion (OLIF) are increasingly replacing traditional approaches. This study aimed to compare the clinical outcomes of OLIF and Mis-TLIF in treating single-level degenerative lumbar diseases.

**Methods:**

Patients with single-level degenerative lumbar diseases underwent either OLIF (30 patients) or Mis-TLIF (30 patients). Surgical data, including operation time, blood loss, postoperative drainage, and postoperative bed rest duration, were collected. Clinical outcomes were assessed using the Oswestry disability index, the visual analog scale scores for low back pain and leg pain, and Japanese Orthopaedic Association scores for daily ability, along with monitoring of complications.

**Results:**

The OLIF group showed significantly shorter operative times, less blood loss, reduced postoperative drainage, and shorter bed rest durations than the Mis-TLIF group. At the 1-month follow-up, OLIF patients also demonstrated significantly better clinical outcome scores than Mis-TLIF patients. No significant differences were observed between OLIF and Mis-TLIF patients before surgery and after 3 months. Furthermore, lumbar lordosis and disc height were significantly greater in the OLIF group at the final follow-up.

**Conclusions:**

Both OLIF and Mis-TLIF achieved satisfactory and effective long-term clinical outcomes for single-level lumbar degenerative diseases. However, OLIF resulted in less tissue damage, reduced bleeding, better short-term clinical outcomes, and improved recovery of segmental lordosis compared to Mis-TLIF. Therefore, OLIF appears to be the preferable option over Mis-TLIF.

## Introduction

Most adults may experience lower back pain (LBP) at least once during their lifetime (1). Lumbar spinal disorders have become one of the most prevalent musculoskeletal diseases. With the rapid aging of the population and advancements in surgical techniques, lumbar interbody fusion has been widely used to treat lumbar spinal disorders. Lumbar interbody fusion has demonstrated proven efficacy for various spinal disorders and enhances arthrodesis, particularly in cases of lumbar degenerative disease (2). The two most established treatments are posterior lumbar interbody fusion (PLIF) and transforaminal lumbar interbody fusion (TLIF). However, with the increasing number of cases and longer follow-up durations, certain disadvantages have continued to emerge, such as iatrogenic muscle injury and residual neurological pain caused by dural adhesion, leading to long-term complications and poor quality of life (3). Therefore, minimally invasive techniques like transforaminal lumbar interbody fusion (Mis-TLIF) and oblique lumbar interbody fusion (OLIF) have been increasingly utilized in the 21st century, gradually replacing traditional approaches (4, 5).

Mis-TLIF has been reported to reduce the iatrogenic soft tissue injury caused by muscle stripping and retraction during spinal exposure (6). Spinal joints and transverse processes are exposed directly through the space between the sacrospinous muscles, which minimizes the retraction of the nerve roots and dural sac (7). In contrast, OLIF reaches the lumbar spine through the large abdominal vascular and the gap between the psoas major muscle, which better avoids nerve structures and allows for inserting a large interbody cage (8). The advantages of the minimally invasive technique include a reduction in muscle trauma, less bleeding and pain, shorter surgical time, and quicker postoperative recovery (8, 9). Nevertheless, the clinical outcomes of the two techniques remain controversial for single-level degenerative lumbar disease. In this study, we aimed to compare the clinical outcomes of OLIF and Mis-TLIF in treating single-level degenerative lumbar diseases.

## Materials and methods

### Patients’ information

We recruited 60 patients with single-level degenerative lumbar disease who have failed conservative treatment and underwent surgery. Patients requiring lumbar interbody fusion for single-segment degenerative disease of the lumbar spine were consecutively enrolled and randomly divided into either the OLIF group (30 patients) or the Mis-TLIF group (30 patients) using a random number table. This study was approved by the institutional review board of the Second People's Hospital of Hefei and performed in strict accordance with the Declaration of Helsinki, Ethical Principles for Medical Research Involving Human Subjects. Informed consent was obtained from all participants.

Before surgery, the abdominal circumference of the patients was measured, and the intestinal tract was cleaned. The instability of the patients’ surgical segment was then comprehensively evaluated. We also assessed the size of each interbody fusion device. Through x-ray, CT, MRI, and other examinations, we observed whether there was yellow ligament ossification, facet joint hyperplasia, or vascular variations in the space between the left psoas major and the abdominal aorta (iliac aorta). Finally, the positions of the left kidney and the left ureter were checked.

### Inclusion and exclusion criteria

The inclusion criteria are as follows: (1) mild lumbar disc herniation, disc-derived LBP, or mild lumbar spondylolysis (Meyerding grade I or II) (10, 11); (2) severe LBP with lower limb radiculopathy symptoms; (3) no improvement after 3 months of systematic conservative treatment; (4) patients undergoing single-segment surgery; (5) symptoms and signs consistent with radiographic findings; and (6) patients with complete clinical and follow-up data.

The exclusion criteria are as follows: (1) multilevel lumbar degenerative disease with apparent symptoms and signs; (2) Meyerding grade II or above lumbar spondylolisthesis (10, 11); (3) previous history of anterior or posterior lumbar surgery; (4) patients with serious diseases such as tumors, infections, or waist fractures; (5) patients with severe medical disorders; (6) patients with severe osteoporosis; (7) previous history of open lumbar surgery; (8) inconsistent clinical symptoms and radiographic data; and (9) absence of a gap between the abdominal vascular sheath and the psoas major muscle at the operative segment.

### Surgical techniques

#### OLIF

(1) The patient was placed in the right lateral decubitus position with a left-sided approach under general anesthesia. The patients’ waist was raised to widen the height of the left intervertebral space. A C-arm machine was used to locate the operating lesion segment and the position of the target intervertebral area, iliac crest, and lower rib margin.

(2) A 3–4 cm longitudinal incision was made 5–9 cm anterior to the midpoint of the target disc.

(3) Blunt dissection was used to separate the external oblique muscle, internal oblique muscle, transverse abdominal muscle, and velum along the direction of the muscle fibers.

(4) A deep retractor was placed to expose the operative field, and a finger or gauze ball was used to gently push the intraperitoneal tissue and extraperitoneal fat forward. Abdominal contents, abdominal vascular sheath, ureter, peritoneum, and other tissues were retracted ventrally by a deep right-angle retractor, which was pressed against the surface of the intervertebral disc. Blunt separation was performed in the front of the psoas major muscle, exposing the raised vertebral space slightly. Careful attention was paid to protecting the ureter, segmental vessels, and sympathetic nerve chain.

(5) Under X-ray fluoroscopy monitoring, a stepwise expansion tube was placed in the first third of the lateral side of the intervertebral space, a working channel of appropriate length was selected to install the free arm, and the light source was set up.

(6) After discectomy, the cartilage endplate was removed using an endplate file and reamer.

(7) An appropriately sized cage was selected to support the intervertebral space, and a fusion device of appropriate size was implanted into the treated intervertebral area.

#### Mis-TLIF

(1) The patients were positioned prone on the spinal operating table under general anesthesia.

(2) Centered on the intersection of the bilateral iliac spine line and the posterior midline of the spine, a longitudinal incision of about 8 cm was made. The paraspinal muscles were dissected laterally along the spinous process to fully expose the facet joint.

(3) The position of the spondylolisthesis was determined by a C-arm machine. Using the “herringbone ridge” as the entry point, two pedicle screws were placed on each side. The targeted vertebra was fixed with long nails. Hemilaminectomy and facetectomy were then performed to decompress the nerve roots.

(4) Discectomy was performed with a bone knife and nucleus pulposus forceps.

(5) The cartilaginous endplate was scraped to the subchondral bone. The intervertebral space was gradually opened with a reamer and an appropriately sized cage.

(6) The bone of the removed lamina and articular process was kept and used to fill the intervertebral space and the lumbar interbody fusion. The interbody fusion device was placed and connected with the posterior screw-rod system.

Specifically, both groups used fusion cage and posterior bilateral nail fixation. After checking that there was no active bleeding in the surgical area and verifying the number of gauze and instruments, the surgical area was repeatedly rinsed with saline. Two drainage tubes were left in place, and the incision was closed layer by layer.

#### Postoperative management

After surgery, patients in both groups received conventional treatment: (1) administering second-generation cephalosporin antibiotics to prevent infection, (2) pain relief, (3) neurotrophic support, and (4) appropriate fluid rehydration. After removal of the intraoperative drainage tube, lumbar anterior and posterior-lateral radiographs were timely reviewed. After postoperative bed rest, patients in both groups wore a barrel brace to get out of bed. Straight leg lifting exercises and lumbar muscle function exercises could be performed after full recovery from postoperative anesthesia. Within 3 months, patients in both groups underwent lumbar back muscle functional training under the protection of lumbar protection. They were advised to avoid excessive sitting and physical labor.

### Clinical outcomes

The researcher who performed the postoperative evaluation was blinded to the patient grouping. Operation time and intraoperative blood loss were recorded and compared between the two groups. The scores of the visual analog scale (VAS) and the Oswestry disability index (ODI) were noted before surgery and at 1 and 3 months postoperatively. Lumbar lordosis (LL), disc height (DH), and other lumbar sagittal parameters were evaluated preoperatively and at the last postoperative follow-up. All patients completed an average of 12.5 months of follow-up. None of the patients experienced spinal nerve root or major blood vessel injury, fusion device displacement, and internal fixation fracture or loosening.

The VAS and the ODI are two commonly used outcome measurement methods in managing spinal disorders. The VAS was used to evaluate pain levels, with scores ranging from 0 to 10, where 0 indicates no pain and 10 indicates the highest level of pain. The ODI was used to assess patients’ functional status and dysfunction. It consists of 10 questions covering pain, self-care, lifting, walking, sitting, standing, sleeping, sex life, social life, and traveling. Each question ranges from 0 to 5, with 0 indicating no symptoms during the activity and 5 indicating that the activity is impossible. The total ODI score is calculated using the following formula: [total score/(5 × number of questions)] × 100 (12). The ODI scale ranges from 0% to 100%. A higher ODI means severe dysfunction.

We also used the Japanese Orthopaedic Association (JOA) assessment to evaluate the degree of leg pain and LBP (13). It includes three parts: cardinal symptoms (LBP, leg pain, and gait), clinical symptoms (straight leg-raising test, sensor dysfunction, and dyskinesia), and activity of daily living (turning over, lifting, cleaning oneself, standing for 1 h, walking, sitting, and forward bending). The total possible score is 29 (poor <10, moderate: 10–15, good: 16–24, optimal: 25–29).

### Statistical analysis

SPSS 25.0 (IBM Corp., Armonk, NY, USA) was used for data analysis. Quantitative variables were presented as means and standard deviations (SDs), while qualitative variables were expressed as numbers and ratios. Student's *t*-test was employed to analyze continuous variables. The categorical variables were analyzed by the chi-square test and the Mann–Whitney test. A two-way ANOVA followed by Tukey's multiple comparisons test was performed to compare various groups. A *P*-value of less than 0.05 was considered statistically significant.

## Results

### General characteristics

The demographics and clinical characteristics of the two groups are presented in Table 1. The mean ages of patients in the OLIF and Mis-TLIF groups were 54.3 ± 10.7 and 56.9 ± 11.4, respectively (*P* = 0.362). There were no significant differences between the two groups in terms of BMI scores (*P* = 0.257 in BMI) and gender distribution (*P* = 0.606). The OLIF group included 10 patients with lumbar intervertebral disc protrusion, 12 patients with lumbar spinal stenosis, and 8 patients with lumbar spondylolisthesis, compared to the corresponding 7, 14, and 9 patients in the Mis-TLIF group (*P* = 0.69).

**Table 1:** Demographics and clinical characteristics of the patients with single-level degenerative lumbar diseases.

	Study group		*P*
	OLIF (*n* = 30)	Mis-TLIF (*n* = 30)	
Age (years)	54.3 ± 10.7 (28–76)	56.9 ± 11.4 (32–75)	0.362
Gender, *n* (%)			
Male	17 (56.7%)	14 (46.7%)	0.606
Female	13 (43.3%)	16 (53.3%)	
BMI (kg/m^2^)	24.4 ± 4.3	23.6 ± 3.9	0.257
Single-level degenerative lumbar diseases, *n* (%)			
Lumbar intervertebral disc protrusion	10 (33.3%)	7 (23.3%)	0.69
Lumbar spinal stenosis	12 (40%)	14 (46.7%)	
Lumbar spondylolisthesis	8 (26.7%)	9 (30%)	
Operation segment (%)			
L_3,4_	6 (20%)	5 (16.7%)	0.999
L_4,5_	24 (80%)	25 (83.3%)	

Values were expressed as *n* (%) or mean ± SD. *P*-values for each group were derived from the chi-square test or the Mann–Whitney test.

BMI, body mass index.

### Intraoperative data

**Figure 1:**
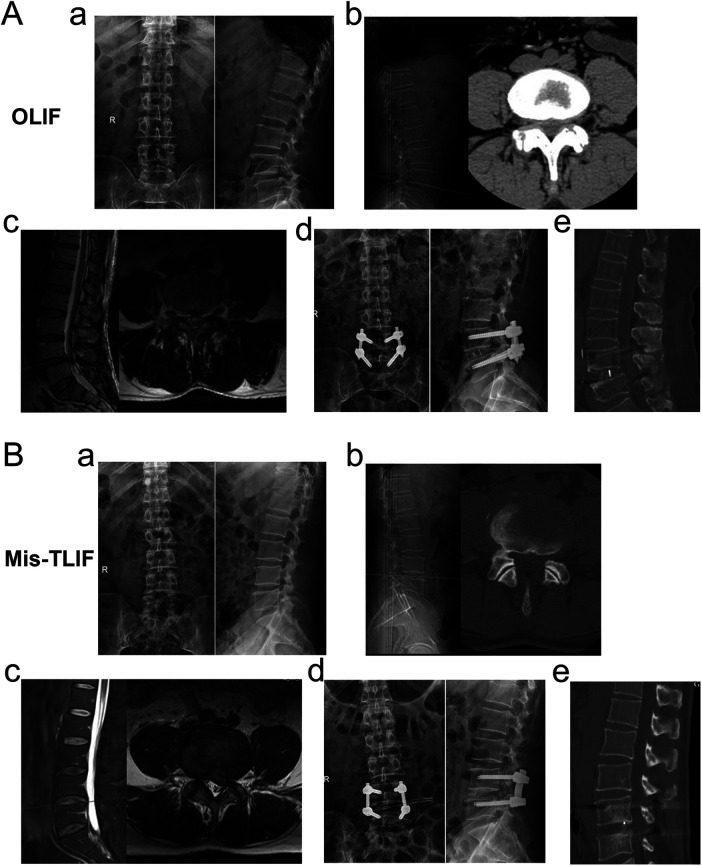
Representative images of patients in the two groups. A 49-year-old male patient who received OLIF in **(A)**. (a) Preoperative anteroposterior and lateral radiographs indicating degenerative changes in the lumbar spine. (b) Preoperative CT indicating a herniated L4/5 intervertebral disc and spinal stenosis. (c) Preoperative MRI indicating L4/5 spinal stenosis. (d) Postoperative anteroposterior and lateral radiographs indicating internal fixation position and suitable vertebral space. (e) CT at the last follow-up showing satisfactory intervertebral height and lumbar lordosis angle and intervertebral bone fusion. A 52-year-old female patient who received Mis-TLIF in **(B)**. (a) Preoperative anteroposterior and lateral radiographs indicating degenerative changes in the lumbar spine. (b) Preoperative CT indicating a herniated L4/5 intervertebral disc and spinal stenosis. (c) Preoperative MRI indicating L4/5 spinal stenosis. (d) Postoperative anteroposterior and lateral radiographs indicating internal fixation position and suitable vertebral space. (e) CT at the last follow-up showing satisfactory intervertebral height and lumbar lordosis angle and intervertebral bone fusion.

**Figure 2:**
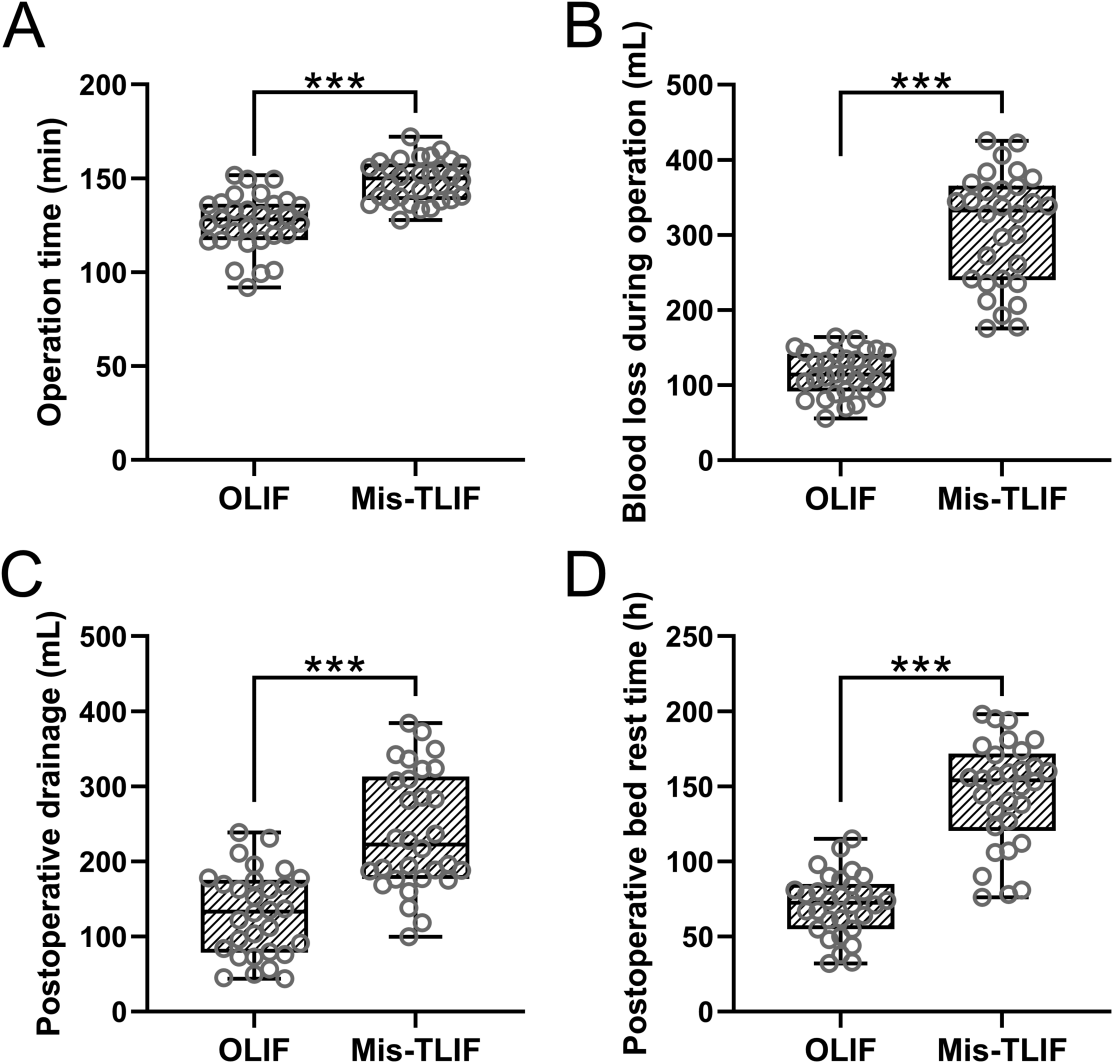
Comparisons of operation time **(A)**, blood loss during operation **(B)**, postoperative drainage **(C)**, and postoperative bed rest duration **(D)** between the patients who underwent OLIF and Mis-TLIF treatments. *N* = 30 for each group. A box plot was used to present the data. ***p < 0.001. Mann–Whitney test was used.

The representative images of two patients in the OLIF and Mis-TLIF groups, taken before surgery, after surgery, and at the last follow-up, are shown in Figures 1A,B. The OLIF group had a significantly shorter average operative time than the Mis-OLIF group (*P* < 0.001, Figure 2A). The OLIF group experienced significantly less blood loss during operation than the Mis-TLIF group (*P* < 0.001, Figure 2B). The average postoperative drainage and bed rest duration were significantly more prolonged in the Mis-TLIF group than in the OLIF group (*P* < 0.001, Figures 2C,D).

### Visual analog scale

**Figure 3:**
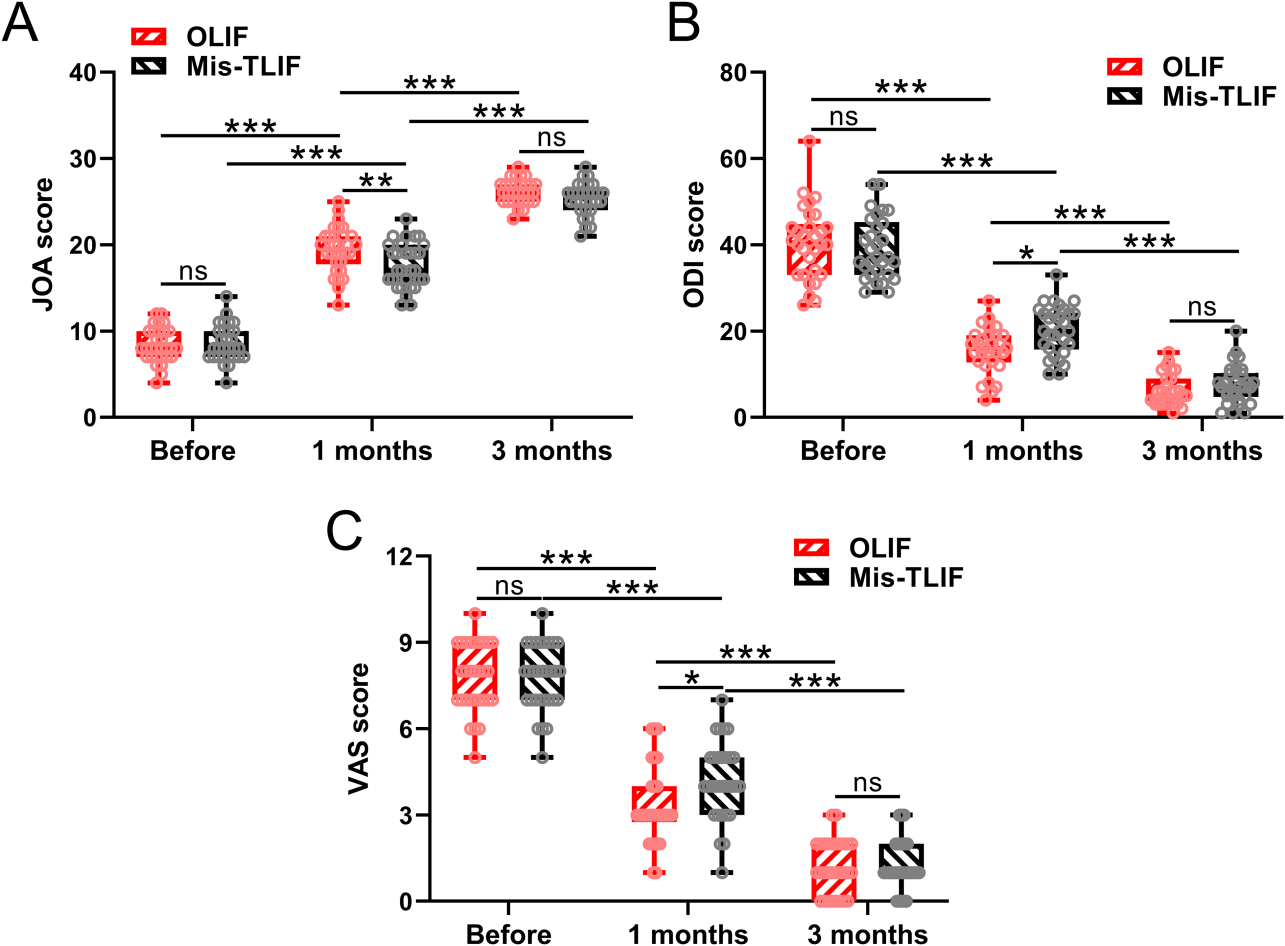
Comparisons of JOA **(A)**, ODI **(B)**, and VAS **(C)** scores between the patients who underwent OLIF and Mis-TLIF treatments. The clinical outcomes were measured before the operation, at 1 month, and at 3 months after the operation. *N* = 30 for each group. A box plot was used to present the data. *p < 0.05; **p < 0.01; ***p < 0.001; ns, no significance. Two-way ANOVA followed by Tukey's multiple comparisons test was used.

Before surgery, there were no significant differences between the two groups in JOA, ODI, and VAS scores. All patients showed significant improvement in outcome scores during the postoperative follow-up compared with their preoperative scores (*P* < 0.001, Figures 3A–C). However, the clinical outcome scores in the OLIF group were significantly better than those in the Mis-TLIF group at 1-month follow-up (*P* < 0.01 in JOA scores, *P* < 0.05 in ODI and VAS scores). After 3 months, there were no significant differences between the two groups in three outcome scores. Thus, the postoperative efficacy of both groups was significantly improved.

### Disc height and lumbar lordosis

**Figure 4:**
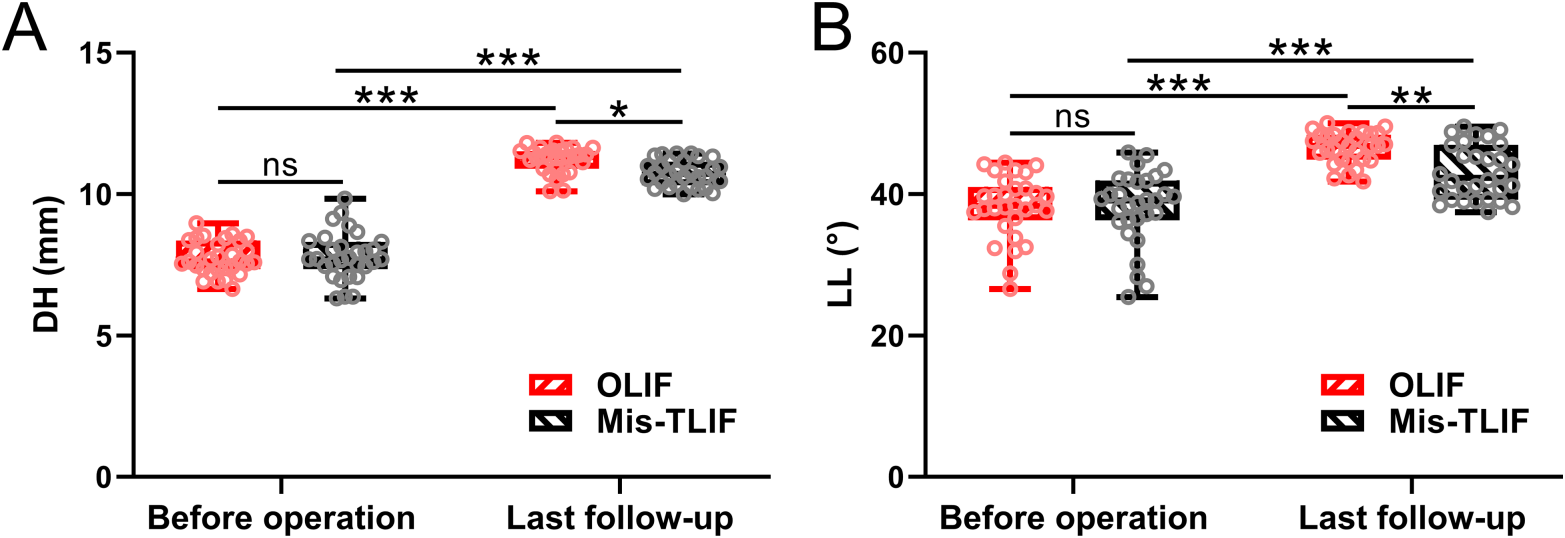
Comparisons of DH **(A)** and LL **(B)** between the patients under the treatment of OLIF and Mis-TLIF. The parameters were measured before the operation and the last follow-up. *N* = 30 for each group. A box plot was used to present the data. *p < 0.05; **p < 0.01; ***p < 0.001; ns, no significance. A two-way ANOVA followed by Tukey's multiple comparisons test was used.

Figure 4 shows the comparison of lumbar sagittal parameters, including DH (Figure 4A) and LL (Figure 4B), between the OLIF and Mis-TLIF groups before the operation and at the last postoperative follow-up. There were no significant differences between the two groups preoperatively. However, at the last follow-up, LL and DH in the OLIF group were found to be significantly higher than in the Mis-TLIF group (*P* < 0.05 in DH, *P* < 0.01 in LL).

## Discussion

LIF techniques have continuously evolved. Minimally invasive techniques such as OLIF and Mis-TLIF offer options for minimized surgical incisions, shorter operation times, reduced blood loss, and quicker recovery phases (14). However, OLIF and Mis-TLIF employ different approaches. OLIF accesses the disc through an anterior corridor between the aorta artery and the psoas muscle in a retroperitoneal plane, reducing postoperative pain by avoiding dissection and splitting of the psoas muscle (15). Despite focusing only on single-level lumbar diseases in this study, OLIF could facilitate access to multiple levels (14). The main disadvantages of OLIF include iatrogenic injury to venous structures and transient weakness with hip flexion (16). If direct spinal decompression is necessary, OLIF may require an additional posterior approach.

In contrast, Mis-TLIF accesses the disc through the posterolateral portion of the foramen with less retraction and a more lateral entry. Therefore, it does not pose a risk to the aorta artery and other major vessels and facilitates direct decompression of neural elements. Mis-TLIF involves less dissection of paraspinal muscle and soft tissues and reduces exposure to midline neural structures (17). However, Mis-TLIF has some disadvantages, such as potential injury to the exiting nerve root and incomplete preparation of the vertebral endplate. In addition, Mis-TLIF only removes one side of the articular process.

Few previous studies have compared OLIF and Mis-TLIF. Lin et al. demonstrated that compared with Mis-TLIF, OLIF had a shorter operative time, less blood loss, higher DH, and earlier fusion time (8, 18). However, Li et al., in a meta-analysis, showed a higher incidence of complications with OLIF than with Mis-TLIF (19). The debate between these two treatments remains controversial. Our study compared the clinical outcomes of OLIF and Mis-TLIF in single-level degenerative lumbar disease. First, we compared the intraoperative data (operation time, blood loss, postoperative drainage, and bed rest duration) between patients who underwent OLIF and Mis-TLIF. Prolonged surgery time might increase the risk of operative complications, while less perioperative blood loss reduces the risk of infection, morbidity, and mortality (5). Our results showed that the OLIF group had significantly shorter operative times, less blood loss, less postoperative drainage, and shorter bed rest durations than the Mis-TLIF group, consistent with previous studies. We also compared postoperative drainage and bed rest duration, and all outcomes indicated that OLIF performed better in surgery data.

Then, we compared the JOA, ODI, and VAS assessments between the two groups. The JOA and VAS scores were used to evaluate levels of the leg and low back pain. ODI was collected to assess patients’ functional status and dysfunction. In previous studies, both OLIF and Mis-TLIF were shown to be effective for degenerative lumbar spondylolisthesis (8). Significant improvements were observed in the ODI and VAS scores (20–22). However, OLIF showed better improvements in postoperative back pain and hospital discharge days (5). Our results demonstrated that the clinical outcome scores of OLIF patients were significantly better than those of Mis-TLIF patients at the 1-month follow-up. There were no significant differences between OLIF and Mis-TLIF groups before surgery and at the 3-month follow-up. This means that OLIF provides better short-term efficiency, while both OLIF and Mis-TLIF are efficient in the long-term follow-up.

Finally, comparisons of lumbar sagittal parameters, including DH and LL, were performed between the OLIF and Mis-TLIF groups. Sagittal balance relates to the alignment of the vertebral spine, and the recovery of DH and LL is associated with postoperative clinical outcomes. In previous studies, patients with restored sagittal balance showed improvements in ODI scores (23), and those undergoing OLIF demonstrated better recovery of segmental lordosis (5). Our results showed that LL and DH were significantly higher in the OLIF group than in the Mis-TLIF group at the last follow-up. A larger and broader cage was implanted into the disc space in the OLIF group compared to the Mis-TLIF group, which may explain why OLIF showed greater improvement in restoring DH and LL. Some studies have suggested that a cage covering the anterior part of the vertebral body could aid in the recovery of segmental lordosis (5, 24, 25). This aligns with our results.

The current study did not take extra steps to control the confounding variables for the following reason: in a randomized controlled trial, randomization ensures the balance of baseline characteristics between the two groups of study participants. Specifically, in our trial, there were no significant differences between the two groups in terms of age, gender, BMI, disease classification, and specific surgical sites. Therefore, the randomization in our trial is a way to control confounding variables, and the data confirm that we have maximally eliminated confounding variables.

However, this study has some limitations. First, the sample size is relatively small, which may introduce selection bias, and the follow-up period is not sufficiently long. Second, the surgical technique was not standardized across all the cases and was performed by only one surgeon. Third, the experience and skills of this surgeon might have influenced the results.

## Conclusion

In conclusion, our results indicate that both OLIF and MIS-TLIF could achieve satisfactory and effective long-term clinical outcomes for single-level lumbar degenerative diseases. However, OLIF exhibited less tissue damage, less bleeding, better short-term clinical outcomes, and better recovery of segmental lordosis. Therefore, OLIF appears to be a better option than Mis-TLIF.

## Data Availability

The raw data supporting the conclusions of this article will be made available by the authors without undue reservation.
